# Ethnicity Moderating the Relationship of Cognition Function of Patients With Dementia on Caregiver Depression

**DOI:** 10.1093/geroni/igaa057.1055

**Published:** 2020-12-16

**Authors:** Alan Palmer, Rowena Gomez, Eric Taylor, Eliot Steer, Megan Frank

**Affiliations:** 1 Palo Alto University, Sunnyvale, California, United States; 2 Palo Alto University, Palo Alto, California, United States

## Abstract

The purpose of this study was to see if ethnicity (African-American and Caucasian) moderates the predictive effects of cognition functioning in patients with dementia on caregivers’ severity of depressive symptoms. Secondary data analyses were conducted from Resources for Enhancing Alzheimer’s Caregiver Health (REACH II; 2001-2004). The participants consisted of 214 African American and 321 Caucasian participants (N = 535). The assessment battery included the Center for Epidemiologic Depression Scale (CES-D) to measure depression severity, Mini-Mental State Exam (MMSE) to measure level of cognitive function, and demographic questionnaire to gain information about caregivers and care-recipients. ANOVAs and ANCOVAs were used to examine ethnic group differences in care-recipient cognitive functioning in predicting caregiver depression. Caucasian caregivers reported significantly higher levels of depression and care-recipients’ cognitive function compared to African American caregivers, ps<.05. A custom ANCOVA indicated a significant interaction between ethnicity and care-recipient cognitive functioning on caregiver depression with greater effects of care-recipient cognitive function on caregiver depression for the African American caregivers than for the Caucasian caregivers, p=.02. Descriptively, the depression severity for the Caucasian caregivers remained relatively high across levels of care-recipients’ cognition. The findings indicated that ethnicity moderated the effects of care-recipient cognitive functioning on caregiver self-report of depressive symptoms. These findings suggest greater resiliency in African -American caregivers supporting their dementia or dementia-related condition care-recipients (Dias et al., 2015). These findings support the need to develop cultural specific interventions to better support the wellbeing of caregivers of care-recipients with dementia or dementia-related conditions.

